# Danish Feasibility Study of a New Innovation for Screening and Brief Intervention for Alcohol Problems in Primary Care: The 15-method

**DOI:** 10.1192/j.eurpsy.2022.2103

**Published:** 2022-09-01

**Authors:** P. Schøler, J. Søndergaard, A. Nielsen

**Affiliations:** 1 The University of Southern Denmark, Unit For Clinical Alcohol Research, Clinical Institute, Odense C, Denmark; 2 The University of Southern Denmark, Research Unit Of General Practice, Department Of Public Health, Odense, Denmark

**Keywords:** Screening and Brief Intervention, Alcohol Treatment, Alcohol use disorder, General Practice

## Abstract

**Introduction:**

The 15-method: a new brief intervention tool for alcohol problems in primary care, has shown promising results in Sweden for mild to moderate alcohol use disorders.

**Objectives:**

To evaluate the 15-method’s usability, organizational integration, and overall implementation feasibility in Danish general practice (GP) in preparation for a large-scale evaluation of the method’s effectiveness in identifying and treating alcohol problems in GP.

**Methods:**

In the Central and Southern Region of Denmark, five general practices participated: seven doctors and eight nurses. Participants received half a day of training in the 15-method. Testing of implementation strategies and overall applicability ran for two months. A focus group interview, two individual interviews with the participating doctors, and five individual patient interviews concluded the study phase.

**Results:**

indicate that implementation of the 15-method is feasible in Danish general practice. The healthcare professionals and patients were optimistic about the method and its possibilities. The method was considered a new patient-centred treatment offer and provided structure to a challenging topic. An interdisciplinary approach was much welcomed. Results indicate that the method is ready for large-scale assessment.

**Conclusions:**

Implementation of the 15-method is considered feasible in Danish general practice, and large-scale evaluation is currently being planned. The results from the present feasibility study, and an overview of the large-scale evaluation, will be presented at the conference.

**Disclosure:**

No significant relationships.

